# The Role of ArlRS and VraSR in Regulating Ceftaroline Hypersusceptibility in Methicillin-Resistant *Staphylococcus aureus*

**DOI:** 10.3390/antibiotics10070821

**Published:** 2021-07-06

**Authors:** Maite Villanueva, Melanie Roch, Iñigo Lasa, Adriana Renzoni, William L. Kelley

**Affiliations:** 1 Department of Microbiology and Molecular Medicine, University Hospital and Medical School of Geneva, 1206 Geneva, Switzerland; maite.villanueva@unavarra.es (M.V.); melanie.roch@unige.ch (M.R.); 2 Departament de Investigación y Desarrollo, Bioinsectis SL, 31110 Noain, Spain; 3 Laboratory of Microbial Pathogenesis, Navarrabiomed, Complejo Hospitalario de Navarra (CHN), Universidad Pública de Navarra (UPNA), 31008 Pamplona, Spain; ilasa@unavarra.es; 4 Service of Infectious Diseases, University Hospital and Medical School of Geneva, 1206 Geneva, Switzerland; adriana.renzoni@hcuge.ch

**Keywords:** ceftaroline, *Staphylococcus aureus*, MRSA, two-component systems, ArlRS, VraSR

## Abstract

Methicillin-resistant *Staphylococcus aureus* infections are a global health problem. New control strategies, including fifth-generation cephalosporins such as ceftaroline, have been developed, however rare sporadic resistance has been reported. Our study aimed to determine whether disruption of two-component environmental signal systems detectably led to enhanced susceptibility to ceftaroline in *S. aureus* CA-MRSA strain MW2 at sub-MIC concentrations where cells normally continue to grow. A collection of sequential mutants in all fifteen *S. aureus* non-essential two-component systems (TCS) was first screened for ceftaroline sub-MIC susceptibility, using the spot population analysis profile method. We discovered a role for both ArlRS and VraSR TCS as determinants responsible for MW2 survival in the presence of sub-MIC ceftaroline. Subsequent analysis showed that dual disruption of both *arlRS* and *vraSR* resulted in a very strong ceftaroline hypersensitivity phenotype. Genetic complementation analysis confirmed these results and further revealed that *arlRS* and *vraSR* likely regulate some common pathway(s) yet to be determined. Our study shows that *S. aureus* uses particular TCS environmental sensing systems for this type of defense and illustrates the proof of principle that if these TCS were inhibited, the efficacy of certain antibiotics might be considerably enhanced.

## 1. Introduction

Infections caused by methicillin-resistant *Staphylococcus aureus* (MRSA) are a major worldwide health problem, including nosocomial and community-acquired infections [1]. Treatment options for MRSA include β-lactams, glycopeptides, and daptomycin, however, resistance to these antibiotics has been reported and in most cases only a few years after their introduction [2]. Therapeutic options now include strict infection control measures, stewardship drug combination regimens, as well as to reliance on recently available antimicrobial agents such as the next-generation cephalosporins (ceftobiprole and ceftaroline) [3].

Ceftaroline possesses broad-spectrum activity against gram-negative and gram-positive bacteria and is highly active against MRSA [4]. Ceftaroline acts by blocking the activity of the principal MRSA transpeptidase PBP2A by triggering its allosteric active site gating that otherwise fails to respond to other β-lactams [5]. Sporadic resistance to ceftaroline has been observed and is associated with mutations in PBP2A itself, but also mutations in *pbp4*, *pbp1*, *pbp2*, *stp1*, *clpX*, *gdpP* but the exact molecular mechanisms are still unknown [6,7,8].

Two-component systems (TCS) are bacterial sensorial systems most often composed of a membrane histidine kinase, which in turn can phosphorylate and activate a cognate intracellular response regulator resulting in changes in gene expression [9]. Most *S. aureus* strains contain 16 TCSs, with one of them being essential for bacterial viability. These proteins are important elements for bacteria to defend from antimicrobials and adapt to changed environmental conditions. More specifically, several TCSs such as *walKR*, *graRS*, *arlRS*, *airRS*, *vraSR,* and *braRS* have some reported implications in cell-wall damage sensing and antibiotic resistance [10,11,12,13].

Studies of antibiotic resistance most commonly involve themes of epidemiologic surveillance, the discovery of resistance mechanisms, or drug efficacy evaluation. Much less attention is devoted to the study of bacterial responses to sub-inhibitory concentrations of antibiotics and the interesting question of whether environmental sensory systems actually contribute a survival advantage in these conditions.

Recently, we described genetically engineered *S. aureus* strains with a form of sensory deprivation [14]. This unique mutant collection represented sequential disruption mutants in all non-essential TCSs of *S. aureus* together with a set of single disruption mutants. These strain sets are invaluable for the study of phenotypes and dissecting environmental sensing pathways.

In this study, we report the use of these TCS disruption strain sets to identify whether, and if so, which TCSs controlled the sub-minimal inhibitory resistance (sub-MIC) to the cell-wall-active antibiotic ceftaroline. Our results suggest that both ArlRS and VraSR TCSs provide cellular protection from sub-MIC ceftaroline and they function in a complementary/cooperative way. Dual disruption of *arlRS/vraSR* shows a dramatic synthetic phenotype and renders cells particularly hypersensitive to the antibiotic. The complementation of either TCS in the total disruption strain is sufficient to sustain growth in the presence of ordinarily sub-inhibitory levels of the antibiotic. This finding reveals how non-essential environmental signal systems may nevertheless govern unforeseen aspects of antibiotic resistance.

## 2. Results

### 2.1. Both vraSR and arlRS TCSs Contribute to Sustain Bacterial Growth in the Presence of Sub-MIC Levels of Ceftaroline

A bacterium needs to detect compounds that act on the cell wall to respond appropriately to antibiotic stress and to develop an adequate resistance response. To identify those sensing systems involved in *S. aureus* sub-inhibitory susceptibility to ceftaroline, we exploited a recently developed collection of TCS mutants [14]. The *S. aureus* genome contains 16 TCSs, among which only WalKR is essential. This collection contains deletion sequential mutants of all TCS present in *S. aureus* MW2 strain, except for the essential *walKR* system. Mutant number ΔI corresponds to deletion of the first *yhscSR* TCS, followed by cumulative mutants in subsequent TCSs (ΔI-ΔXV). As an example, mutant number ΔXI corresponds to deletion of 11 TCSs including *vraSR*, but still containing *phoRP*, *arlRS*, *agrBDCA*, *srrAB*, and *walKR*. Finally, the MW2 ΔXV strain lacks all non-essential TCSs but maintains *walKR* in its genome.

**Figure 1:**
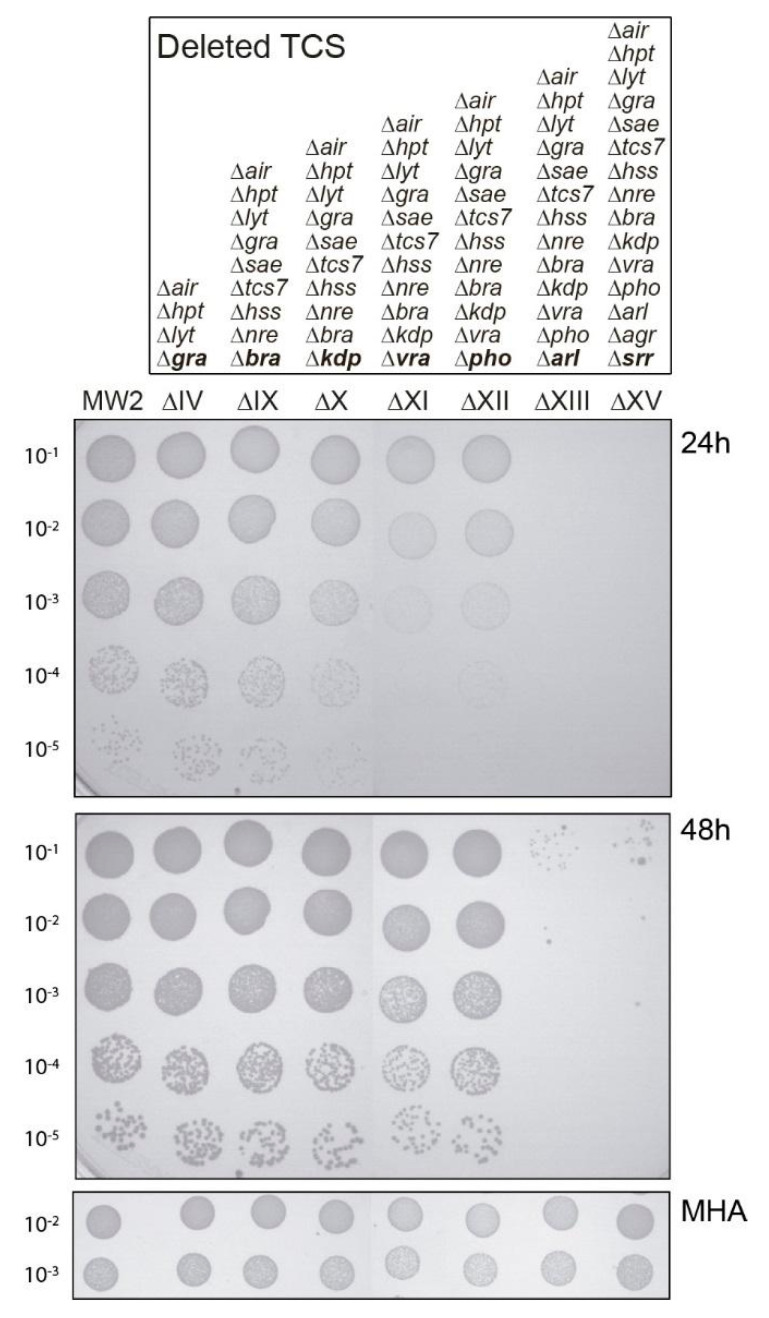
Effect of TCS deletions on enhanced ceftaroline susceptibility. Spot plating population analysis profiles (Spot PAP) of MW2 and their corresponding ΔIV, ΔIX, ΔX, ΔXI, ΔXII, ΔXIII, and ΔXV mutant strains on MHA plates containing ceftaroline. The deleted TCS in each strain is indicated in bold. Upper panels correspond to MHA plates containing 0.25 µg/mL of ceftaroline at 24 and 48 h of incubation, respectively. The lower panel corresponds to control MHA plates without antibiotics. Spot serial 10-fold dilutions are indicated at the left margin. The first spot 10 µL corresponds to 1.5 × 10^5^ colony forming units (CFU).

The ceftaroline susceptibility profile of all 15 sequential TCS mutants in strain MW2 were tested by spot test analysis on ceftaroline supplemented agar plates. As shown in Figure 1, after 24 h of incubation no changes in susceptibility were observed for mutants ΔI to ΔX, suggesting that the majority of the TCSs were not involved in the tested hypersensitivity response to ceftaroline, excluding *vraSR*, *phoRP*, *arlRS*, *agrBDCA*, *srrAB,* and *walKR*. After 24 h incubation, a somewhat higher susceptibility to ceftaroline was observed when deleting *vraSR*. No changes were observed with subsequent *phoRP* deletion, but an increased susceptibility was observed with *arlRS* mutation and maintained following *agrCA* and *srrAB* deletions (Figure 1). These results suggested that mutations in the TCSs *vraSR* and *arlRS* were implicated in increased susceptibility to ceftaroline. The increased susceptibility observed in the *arlRS* mutation, after removing *vraSR*, suggested a complementary/cooperative effect of both TCSs on the ceftaroline enhanced susceptibility phenotype. After 48 h of incubation, the apparent increased sensitivity to ceftaroline caused by the lack of *vraSR* disappeared, probably because the strain still retained *arlRS* (Figure 1).

The deletion progression from MW2 XVIII to XV includes disruptions of the AgrCA and SrrAB TCS systems. To exclude a role for these two TCS systems in the observed ceftaroline hypersensitivity apparently linked with *vraSR* and *arlRS* disruption, we performed spot test analysis using MW2 with single deletions of *srrAB* or *agrCA* as well as chromosomally complemented ΔXIV deletion strains (Supplementary Figure S1). No role in modulating ceftaroline hypersensitivity was detected by this analysis for either *srrAB* or *agrBDCA* TCS system.

### 2.2. Analysis of arlRS and vraSR Single and Double Mutation

**Figure 2:**
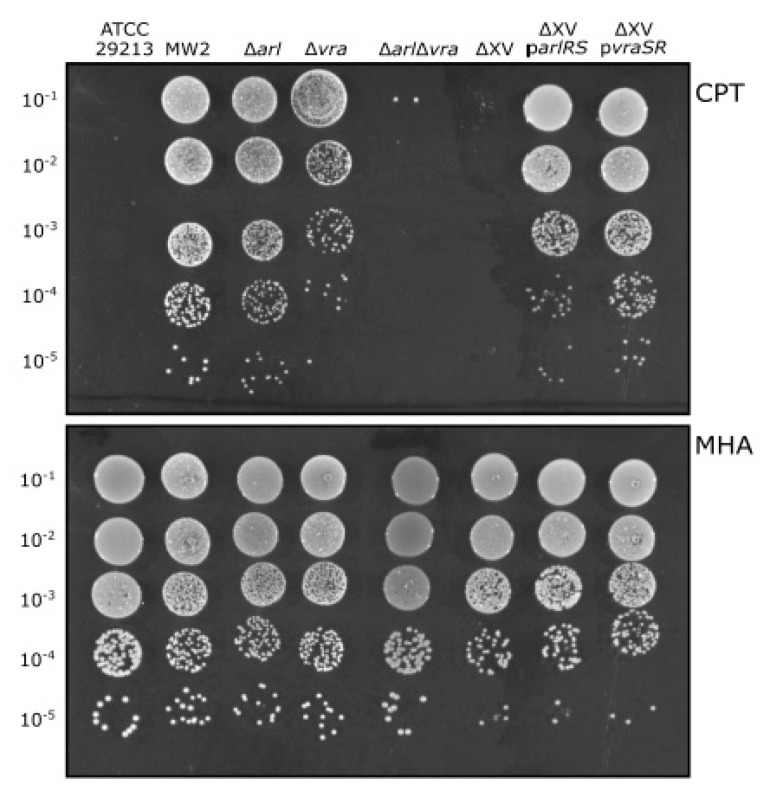
The role of *arlRS* and *vraSR* TCS disruption on enhanced ceftaroline susceptibility. Spot plating population analysis profiles (Spot PAP) of MW2 and the corresponding *arlRS* or/and *vraSR*-deleted strains together with ΔXV strain and ΔXV complemented with either the indicated *arlRS*- or *vraSR*-expressing plasmids on MHA plates. ATCC29213 was used as the quality control standard strain. The upper panel corresponds to MHA plates containing 0.25 µg/mL of ceftaroline (CPT) at 48 h. The lower panel corresponds to MHA control plates without ceftaroline. Spot serial 10-fold dilutions are indicated at the left margin. The first spot (10 µL) corresponds to 1.5 × 10^5^ colony forming units (CFU).

To confirm the role of VraSR and ArlRS in the enhanced ceftaroline susceptibility profile, *vraSR*, *arlRS,* or both were individually deleted in *S. aureus* MW2. Mutation of *arlRS* alone did not show any effect on ceftaroline susceptibility; however, the *vraSR* mutant showed reduced growth on ceftaroline. These results strongly suggest that VraSR can compensate for the absence of ArlRS (Figure 2 and Table 1). Consistent with this hypothesis, the double *vraSR/arlRS* deletion showed a dramatically enhanced ceftaroline susceptibility, both in PAP and ECF, demonstrating that both TCSs are involved in the drug response and supporting the notion of a complementary/cooperative function (Figure 2 and Table 1). This defective growth phenotype was slightly reflected in the MIC/MCB.

To prove genetic causality, the MW2 ΔXV strain, lacking all non-essential TCSs, was next complemented with plasmids carrying the *vraSR* or *arlRS* operons under the control of the heterologous cadmium-inducible plasmid promoter without cadmium (just its leaky expression). Exogenous expression of *arlRS* or *vraSR* systems in a strain lacking all other TCSs showed remarkably that either system was sufficient to counteract enhanced ceftaroline susceptibility. We observed that the strain harboring p*vraSR* was able to grow better than the strain harboring p*arlRS* in these experimental conditions as judged by colony-forming assay (Figure 2) and the MIC/MBC values (Table 1). The mechanistic basis underlying this observation is presently unknown.

**Figure 3:**
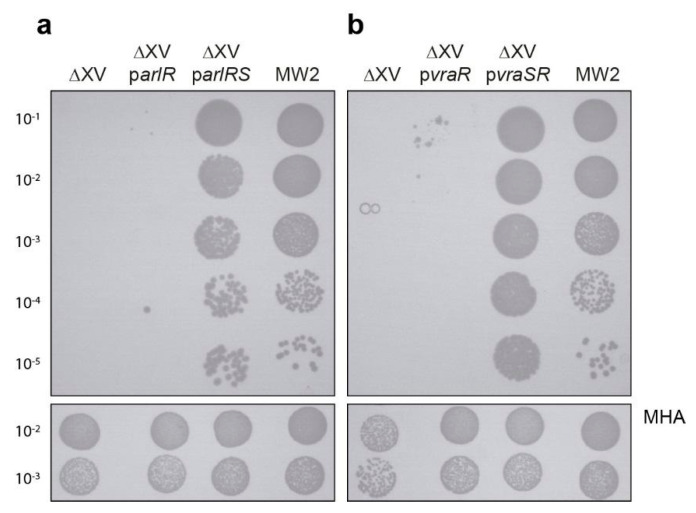
(**a**) *arl* and (**b**) *vra* histidine kinases are necessary for ceftaroline response. Spot plating population analysis profiles (Spot PAP) of MW2, ΔXV, and ΔXV strain complemented with *arl* and *vra* response regulators alone or the complete TCS, on MHA plates containing ceftaroline at 48 h. The upper panel corresponds to MHA plates containing 0.25 µg/mL of ceftaroline. The lower panel corresponds to MHA plates without ceftaroline. Spot serial 10-fold dilutions are indicated at the left margin. The first spot (10 µL) corresponds to 1.5 × 10^5^ colony forming units (CFU).

In the absence of the cognate membrane sensor component, the expression of the *vraR* or *arlR* transcriptional regulators alone was not sufficient to modify the susceptibility to ceftaroline, suggesting that both sensors were crucial for transcriptional regulators to be phosphorylated and thus activate the pathways modulating the cellular response to ceftaroline (Figure 3).

### 2.3. Analysis of Ceftaroline Sensitivity by Early Time Kill Assay

**Figure 4:**
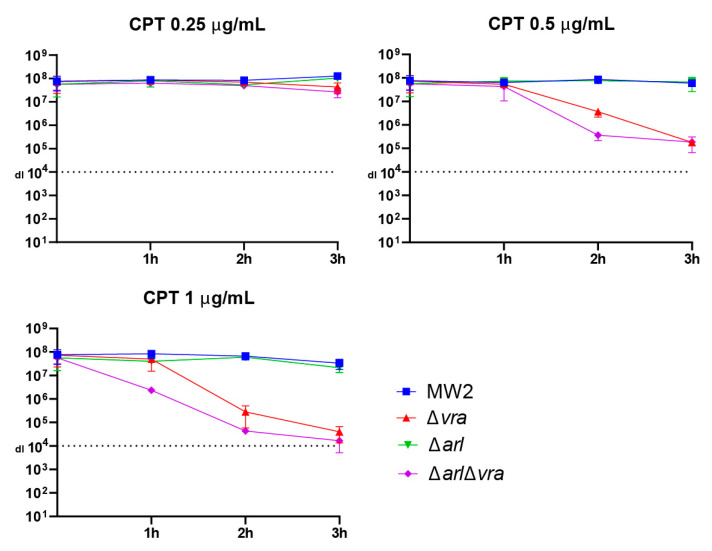
Early time-kill assay. The indicated four CA-MRSA MW2 strains and derivatives, all with equivalent MIC, were exposed to three concentrations (in μg/mL) of ceftaroline (CPT: 1/2 MIC, MIC, and 2 × MIC) in broth at 37 °C (Materials and Methods). Aliquots were removed every hour and viable cell titers were determined by serial dilution and plating on MHA plates without drug. The data represent the results of three biologically independent determinations and curves are plotted ± SD. Note that for CPT 0.5 and 1.0 conditions, the double *arlRS/vraSR* mutant consistently reduces viable cell titer faster than the single *vraSR* mutant strain.

To determine whether the hypersensitivity to ceftaroline in the double mutant *arlRS*/*vraSR* strain observed on agar plate assay was demonstrable in a broth assay, we performed early time-kill experiments. We tested the response of MW2, and its single or double mutant derivatives: Δ*arlRS*, Δ*vraSR*, and Δ*arlRS*/Δ*vraSR,* to challenge with ceftaroline in a concentration range 0.25 μg/mL to 1μg/mL (1/2 MIC, MIC, and 2 × MIC). Aliquots were removed over a three-hour interval, serially diluted, and spotted on MHA plates without drug to measure viable titers. The results are plotted in Figure 4. Whereas little difference was observed for strains exposed to 1/2 MIC over this time interval, we clearly observed that the Δ*arlRS*/Δ*vraSR* strain showed greater reduced viable cell counts compared to Δ*vraSR* in the other two conditions. We did not observe any change in the time-kill of the Δ*arlRS* mutant compared to the MW2 wild-type control. We conclude from this analysis that the double mutant Δ*arlRS*/Δ*vraSR* strain is more sensitive to ceftaroline challenge, especially when drug concentrations are in the clinically relevant range. Taken together, the agar plate assay and time-kill assay show that dual disruption of the two TCS sensory systems *arl* and *vra* imparts hypersensitivity to ceftaroline.

## 3. Discussion

We report the implication of disruption of two particular non-essential TCSs affecting the enhanced susceptibility to ceftaroline of *S. aureus* CA-MRSA strain MW2 by taking advantage of a previously generated battery of sequential mutants in *S. aureus* TCSs [14]. In this work, we discovered that disruption of both *arlRS* and *vraSR* TCSs resulted in a strong phenotype and demonstrated that they are implicated in the maintenance of MW2 growth in the presence of sub-MIC levels of ceftaroline. Although both TCSs appear to contribute cooperatively to this type of sub-MIC ceftaroline resistance, complementation with a multicopy plasmid encoding VraSR alone is sufficient to completely restore the prior resistance profile to ceftaroline of the *S. aureus* ΔXV strain. In contrast, complementation with p*arlRS* restores only partially the phenotype. These results may be explained because the regulons of both TCS might partially overlap [15]. Thus, when VraSR is active all the genes necessary to respond for the modifications in the cell wall homeostasis will be activated whereas, in contrast, only part of the required genes would be activated by ArlRS. A precise characterization of the regulons of each TCS will be necessary to answer this question.

A role for VraSR in ceftaroline susceptibility for the strain MW2 is not unexpected since VraSR is a positive regulator of cell-wall peptidoglycan synthesis and is deeply involved in β-lactam and glycopeptide resistance [12,16]. VraSR also directly regulates genes such as extracellular protein folding and quality control factors *prsA* and *htrA1*, which are necessary to sustain PBP2a biogenesis and β-lactam resistance in MRSA strains [17]. This latter finding is especially relevant to the present study since ceftaroline’s mode of action involves its interaction with PBP2a and disrupting the allosteric mechanism to allow active site gating and active site serine 403 acylation [5]. Thus, a failure to upregulate the VraR-dependent transcription of *prsA* and *htrA1* upon cell wall stress culminates in less PBP2a available because of impaired post-translational secretion maturation [17]. Such a scenario could conceivably result in ceftaroline hypersensitivity based upon a reduced target concentration.

Disruption of *vra* also leads to a pronounced Triton X-100 hypersensitivity suggesting that overall cell membrane integrity might be compromised in this strain [14]. None of the other reported TCS disruptions displayed this Triton sensitivity phenotype [14]. Taken together, these findings suggest the VraRS contribution to ceftaroline hypersensitivity is likely multifactorial.

Our study also revealed that ArlRS has an important role in the response to ceftaroline in our experimental system. ArlRS has been described as a global regulator of *S. aureus* virulence, extracellular proteases, capsule formation, and is a direct regulator of *mgrA* [18]. The extensive and complex ArlRS regulon, which displays a 70% overlap with the redox-sensitive MgrA regulon [19,20], indicates that it might be difficult to pinpoint a particular ArlRS-dependent function that precisely explains our observed ceftaroline hypersensitivity. Nevertheless, a predicted ArlR binding site was shown in the Spx promoter region. Importantly, recent investigations have revealed that ArlRS has a role in oxacillin susceptibility through its regulation of the global stress regulator Spx [10]. Spx is essential in *S. aureus* and controls a number of genes involved in oxidative stress and the maintenance of redox homeostasis [21,22]. Processes regulated by Spx may therefore also contribute to antibiotic susceptibility defense mechanisms.

How precisely ArlRS and VraSR TCSs coordinately contribute to cellular defense against sub-inhibitory levels of ceftaroline will be important to elucidate. Since many naturally occurring antibiotics and antibiotic resistance genes are thought to have originated as signaling molecules and/or contribute to complex bacterial population dynamics [23], it is tempting to speculate that a number of metabolic processes arose to counteract these anti-microbial molecules encountered in very low (sub-therapeutic) concentrations. Uncovering these pathways and discovering a means to inhibit them should constitute a particularly viable adjuvant strategy to augment the therapeutic efficacy of antimicrobials.

Indeed, in recent years, two-component signal transduction systems have been shown to be important targets in the antibacterial fight since their histidine phosphorylation differs from normal serine/threonine and tyrosine phosphorylation in higher eukaryotes. Drugs that target TCSs could be highly effective not only because it affects specific essential functions, but also because it impairs upstream regulatory functions related to the physiology of the pathogen [24]. Therefore, the use of TCSs for drug development provides an alternative approach for combating microbial infections, including those caused by antibiotic-resistant pathogens.

## 4. Materials and Methods

### 4.1. Bacterial Strains and Culture Conditions

Strains and plasmids used in this study are listed in Table 2. *Escherichia coli* strains were grown in Luria-Bertani broth (LB, BD/Difco, Basel, Switzerland) and *Staphylococcus aureus* strains were grown in Mueller-Hinton broth (MHB, BD/Difco, Basel, Switzerland). When required for growth or selection, the medium was supplemented with appropriate antibiotics at the following concentrations: ampicillin, 100 μg/mL; and erythromycin, 1.5 and 10 μg/mL. Recombinant lysostaphin was obtained from AMBI Products LLC (Lawrence, NY, USA). The pCN51 inducible plasmid [25] shows a basal expression in the absence of cadmium. All the experiments performed in this study that involve the pCN51 plasmid were carried out without cadmium supplementation.

### 4.2. Electrocompetent Staphylococcus Cells

Staphylococcal electrocompetent cells were generated as previously described [26]. Briefly, bacteria were grown in 200 mL of B2 broth at 37 °C with shaking (200 rpm) until an OD_600_ of 0.5. Cultures were incubated on ice (15 min) and then harvested and the pellet washed three times with sterile water. A final washing was done with 30 mL of ice-cold 10% (*v*/*v*) glycerol. The pellet was resuspended into 15 mL of ice-cold 10% glycerol and incubated for 15 min at 20 °C. Cultures were centrifuged and pellets resuspended with 200 μL of ice-cold 10% glycerol. Aliquots (50 μL) were stored at −80 °C. Plasmids were transformed into staphylococci by electroporation as previously described [27].

### 4.3. Construction of MW2 Δarl Δvra Strain

pMAD::TCS12AD plasmid [28] was purified from *S. aureus* RN4220 and then transformed into *S. aureus* MW2 Δarl strain by electroporation. Homologous recombination experiments were performed as described [14]. Erythromycin-sensitive white colonies, which did not further contain the pMAD plasmid, were verified by PCR assay using primers vra-E (TGACGAACAAGTGAAATGG) and vra-F (CGTTCTATTATTGGGATGTG).

### 4.4. Spot Test Assay

The spot population analysis profile (PAP) method was used to assess antibiotic resistance within the population, as previously described [29]. *S. aureus* overnight cultures, supplemented with erythromycin for plasmid selection, were adjusted to a 0.5 McFarland standard (1.5 × 10^8^ bacteria/mL), corresponding to an OD_600_ of 0.1 using a turbidity Densimat apparatus (bioMerieux, Marcy-L’Etoile, France). Serial 10-fold dilutions (10^−1^ to 10^−5^) were prepared, and then aliquots (10 µL) of each dilution were spotted on freshly prepared MH agar (MHA) plates containing 0.25 μg/mL of ceftaroline. MHA plates without ceftaroline were used as control. Viable colonies were examined after 24 and 48 h incubation at 37 °C. The results reported were consistent across at least three independent assays. The relative efficiency of colony formation (ECF) was calculated by normalizing the number of colonies, scored on plates containing antibiotic at 48 h, to the number of bacteria obtained on agar without antibiotics.

### 4.5. Antibiotic Susceptibility Tests

Broth microdilution MICs were performed according to EUCAST (European Commission on Antibiotic Susceptibility Testing) guidelines in a 96-well microplate in MHB, as previously described [16]. Briefly, a 0.5 McFarland standard cell suspension was prepared from a 24 h agar culture in NaCl 0.9% using a bioMérieux Densimat apparatus (bioMérieux, France). After 1:100 dilution in MHB, 50 µL was added to 50 µL of 2× ceftaroline solution to obtain a final concentration range from 0.125 to 8 μg/mL. Microplates were incubated for 24 h at 37 °C. *S. aureus* MSSA (methicillin-sensitive) strain ATCC 29213 was used as a standard reference quality control. Determinations were performed in triplicate assay and the composite data reported as the modal value together with the range from a minimum of three independent biological determinations. For MBC (minimum bactericidal concentration) calculation 10 µL of each well was plated in MHB. Early time-kill assays were performed essentially using the protocol for MIC microdilution assay described above in a 200 μL volume with the indicated final ceftaroline concentration. Aliquots (20 μL) were removed at the indicated time points and serially 10-fold diluted in MHB and then aliquots (10 μL) of 10^−1^ to 10^−5^ dilutions were spotted on MHA plates without drug. The data from three independent biological determinations were compiled in GraphPad for display.

## Figures and Tables

**Table 1:** ECF ^a^ on 0.25 µg/mL of ceftaroline-supplemented agar and MIC/MBC.

Strains	ECF ^a^ with Ceftaroline at 0.25 µg/mL	Modal MIC (Range)	Modal MBC (Range)
MW2	≈1	0.5 (0.5–1)	1
Δ*arl*	≈1	0.5	1 (0.5–1)
Δ*vra*	2 × 10^−2^	0.5 (0.5–1)	0.5
Δ*arl* Δ*vra* ^b^	<10^−8^	0.5 (0.25–0.5)	0.5
ΔXV ^b^	<10^−8^	0.25 (0.25–0.5)	0.5
ΔXV p*arlRS*	2 × 10^−1^	0.5	0.5
ΔXV p*vraSR*	≈1	1 (0.5–1)	1 (0.5–1)
ATCC29213	<10^−8^	0.5 (0.25–0.5)	0.5

^a^ The number of survivors in the presence of ceftaroline was normalized to the number of bacteria plated on agar without ceftaroline. Data are reported for at least three independent experiments; ^b^ Strains showing no single CFU on agar supplemented with ceftaroline at a concentration of 0.25 µg/mL. The viable counts on agar without ceftaroline were >10^8^ CFU/mL.

**Table 2:** Plasmids and strains were used in this study.

**Plasmids**	**Relevant Characteristics**	**Reference**
pMAD::TCS12AD	pMAD plasmid containing the allele for deletion of the *vraSR* genes	[14]
p*arlRS*	pCN51 plasmid expressing *arlRS* genes	[14]
p*vraSR*	pCN51 plasmid expressing *vraSR* genes	[14]
p*arlR*	pCN51 plasmid expressing *arlR* gene	[14]
p*vraR*	pCN51 plasmid expressing *vraR* gene	[14]
**Strains**	**Relevant Characteristics**	**Reference**
ATCC29213	Standard QC strain MSSA	
MW2	Typical community-acquired strain of MRSA, which was isolated in 1998 in North Dakota, USA. *bla*^+^ Δ*mecR1 mecI*^−^ *mecR2*^−^	[14]
ΔIV	MW2 ∆*airSR*, ∆*hptSR,* ∆*lytSR,* ∆*graRS*	[14]
ΔIX	MW2 ∆*airSR*, ∆*hptSR,* ∆*lytSR,* ∆*graRS,* ∆*saeRS*, ∆*tcs7,* ∆*hssRS,* ∆*nreBC,* ∆braRS	[14]
ΔX	MW2 ∆IX ∆*kdpDE*	[14]
ΔXI	MW2 ∆X ∆*vraSR*	[14]
ΔXII	MW2 ∆XI ∆*phoPR*	[14]
ΔXIII	MW2 ∆XII ∆*arlRS*	[14]
ΔXV	MW2 ∆XIII ∆*agrCA,* ∆*srrAB*	[14]
ΔXIV (*srrAB*)	MW2 ∆XIII ∆*agrCA*	[14]
ΔXIV (*agrBDCA*)	MW2 ∆XIII ∆*srrAB*	[14]
Δ*srr*	MW2 ∆*srrAB*	[14]
Δ*agr*	MW2 ∆*agrCA*	[14]
Δ*arl*	MW2 ∆*arlRS*	[14]
Δ*vra*	MW2 ∆*vraSR*	[14]
Δ*arl* Δ*vra*	MW2 ∆*arlRS* ∆*vraSR*	This study
ΔXV p*arlRS*	MW2 ∆XV ∆*arl* carrying pCN51::*arlRS* plasmid	[14]
ΔXV p*vraSR*	MW2 ∆XV ∆*arl* carrying pCN51::*vrASR* plasmid	[14]
ΔXV p*arlR*	MW2 ∆XV carrying pCN51::*arlR* plasmid	[14]
ΔXV p*vraR*	MW2 ∆XV carrying pCN51::*vraR* plasmid	[14]

## Data Availability

All included in manuscript text.

## Supplementary Materials

The following are available online at https://www.mdpi.com/article/10.3390/antibiotics10070821/s1, Figure S1: Spot test assay for sub-MIC ceftaroline sensitivity.

## References

[1] Lowy F.D. (1998). Staphylococcus aureus infections. N. Engl. J. Med..

[2] Chambers H.F., DeLeo F.R. (2009). Waves of resistance: *Staphylococcus aureus* in the antibiotic era. Nat. Rev. Microbiol..

[3] Purrello S.M., Garau J., Giamarellos E., Mazzei T., Pea F., Soriano A., Stefani S. (2016). Methicillin-resistant *Staphylococcus aureus* infections: A review of the currently available treatment options. J. Glob. Antimicrob. Resist..

[4] Jacqueline C., Caillon J., Le Mabecque V., Miègeville A.F., Hamel A., Bugnon D., Ge J.Y., Potel G. (2007). In vivo efficacy of ceftaroline (PPI-0903), a new broad-spectrum cephalosporin, compared with linezolid and vancomycin against methicillin-resistant and vancomycin-intermediate *Staphylococcus aureus* in a rabbit endocarditis model. Antimicrob. Agents Chemother..

[5] Fishovitz J., Rojas-Altuve A., Otero L.H., Dawley M., Carrasco-López C., Chang M., Hermoso J.A., Mobashery S. (2014). Disruption of allosteric response as an unprecedented mechanism of resistance to antibiotics. J. Am. Chem. Soc..

[6] Long S.W., Olsen R.J., Mehta S.C., Palzkill T., Cernoch P.L., Perez K.K., Musick W.L., Rosato A.E., Musser M. (2014). PBP2a mutations causing high-level ceftaroline resistance in clinical methicillin-resistant *Staphylococcus aureus* isolates. Antimicrob. Agents Chemother..

[7] Andrey D.O., François P., Manzano C., Bonetti E.J., Harbarth S. (2017). Antimicrobial activity of ceftaroline against methicillin-resistant *Staphylococcus aureus* (MRSA) isolates collected in 2013–2014 at the Geneva University Hospitals. Eur. J. Clin. Microbiol. Infect. Dis..

[8] Varela M.C., Roch M., Taglialegna A., Long S.W., Saavedra M.O., Rose W.E., Davis J.J., Hoffman L.R., Hernandez R.E., Rosato R.R. (2020). Carbapenems drive the collateral resistance to ceftaroline in cystic fibrosis patients with MRSA. Commun. Biol..

[9] Gao R., Bouillet S., Stock A.M. (2019). Structural basis of response regulator function. Annu. Rev. Microbiol..

[10] Bai J., Zhu X., Zhao K., Yan Y., Xu T., Wang J., Huang W., Shi L., Shang Y., Lv Z. (2019). The role of ArlRS in regulating oxacillin susceptibility in methicillin-resistant *Staphylococcus aureus* indicates it is a potential target for antimicrobial resistance breakers. Emerg. Microbes Infect..

[11] Cardona S.T., Choy M., Hogan A.M. (2017). Essential two-component systems regulating cell envelope functions: Opportunities for novel antibiotic therapies. J. Membr. Biol..

[12] Kuroda M., Kuroda H., Oshima T., Takeuchi F., Mori H., Hiramatsu K. (2003). Two-component system VraSR positively modulates the regulation of cell-wall biosynthesis pathway in *Staphylococcus aureus*. Mol. Microbiol..

[13] Rapun-Araiz B., Haag A.F., Solano C., Lasa I. (2020). The impact of two-component sensorial network in staphylococcal speciation. Curr. Opin. Microbiol..

[14] Villanueva M., García B., Valle J., Rapún B., Ruiz De Los Mozos I., Solano C., Martí M., Penadés J.R., Toledo-Arana A., Lasa I. (2018). Sensory deprivation in *Staphylococcus aureus*. Nat. Commun..

[15] Rapun-Araiz B., Haag A.F., De Cesare V., Gil C., Dorado-Morales P., Penades J.R., Lasa I. (2020). Systematic Reconstruction of the Complete Two-Component Sensorial Network in *Staphylococcus aureus*. Msystems.

[16] Galbusera E., Renzoni A., Andrey D.O., Monod A., Barras C., Tortora P., Polissi A., Kelley W.L. (2011). Site-specific mutation of *Staphylococcus aureus* VraS reveals a crucial role for the VraR-VraS sensor in the emergence of glycopeptide resistance. Antimicrob. Agents Chemother..

[17] Roch M., Lelong E., Panasenko O.O., Sierra R., Renzoni A., Kelley W.L. (2019). Thermosensitive PBP2a requires extracellular folding factors PrsA and HtrA1 for *Staphylococcus aureus* MRSA B-lactam resistance. Commun. Biol..

[18] Crosby H.A., Tiwari N., Kwiecinski J.M., Xu Z., Dykstra A., Jenul C., Fuentes E.J., Horswill A.R. (2020). The *Staphylococcus aureus* ArlRS two-component system regulates virulence factor expression through MgrA. Mol. Microbiol..

[19] Linzner N., Van Loi V., Fritsch V.N., Antelmann H. (2021). Thiol-based redox switches in the major pathogen *Staphylococcus aureus*. Biol. Chem..

[20] Chen P.R., Bae T., Williams W.A., Duguid E.M., Rice P.A., Schneewind O., He C. (2006). An oxidation-sensing mechanism is used by the global regulator MgrA in *Staphylococcus aureus*. Nat. Chem. Biol..

[21] Villanueva M., Jousselin A., Baek K.T., Prados J., Andrey D.O., Renzoni A., Ingmer H., Frees D., Kelley W.L. (2016). Rifampin resistance *rpoB* alleles or multicopy thioredoxin/thioredoxin reductase suppresses the lethality of disruption of the global stress regulator *spx* in *Staphylococcus aureus*. J. Bacteriol..

[22] Pamp S.J., Frees D., Engelmann S., Hecker M., Ingmer H. (2006). Spx is a global effector impacting stress tolerance and biofilm formation in *Staphylococcus aureus*. J. Bacteriol..

[23] Allen H.K., Donato J., Wang H.H., Cloud-Hansen K.A., Davies J., Handelsman J. (2010). Call of the wild: Antibiotic resistance genes in natural environments. Nat. Rev. Microbiol..

[24] Gotoh Y., Eguchi Y., Watanabe T., Okamoto S., Doi A., Utsumi R. (2010). Two-component signal transduction as potential drug targets in pathogenic bacteria. Curr. Opin. Microbiol..

[25] Charpentier E., Anton A.I., Barry P., Alfonso B., Fang Y., Novick R.P. (2004). Novel cassette-based shuttle vector system for Gram-positive bacteria. Appl. Environ. Microbiol..

[26] Schenk S., Laddaga R.A. (1992). Improved method for electroporation of *Staphylococcus aureus*. FEMS Microbiol. Lett..

[27] Cucarella C., Solano C., Valle J., Amorena B., Lasa I.G.O., Penade R. (2001). Bap, a *Staphylococcus aureus* surface protein involved in biofilm formation. J. Bacteriol..

[28] Toledo-Arana A., Merino N., Vergara-Irigaray M., Débarbouillé M., Penadés J.R., Lasa I. (2005). *Staphylococcus aureus* develops an alternative, ica-independent biofilm in the absence of the *arlRS* two-component system. J. Bacteriol..

[29] Renzoni A., Andrey D.O., Jousselin A., Barras C., Monod A., Vaudaux P., Lew D., Kelley W.L. (2011). Whole genome sequencing and complete genetic analysis reveals novel pathways to glycopeptide resistance in *Staphylococcus aureus*. PLoS ONE.

