# Supplementary material for: The Role of ArlRS and VraSR in Regulating Ceftaroline Hypersusceptibility in Methicillin-Resistant Staphylococcus aureus
Source: Antibiotics (Basel). 2021 Jul 6;10(7):821. doi: 10.3390/antibiotics10070821 (PMC8300640; doi:10.3390/antibiotics10070821)
Supplement: Supplementary file 1 [file antibiotics-10-00821-s001.zip › antibiotics-1099082-supplementary.pdf]

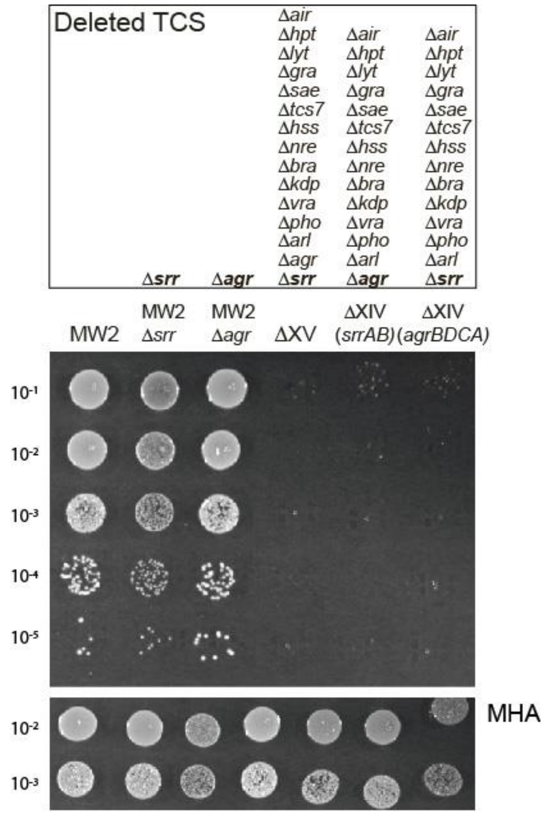

Supplemental Figure S1. Spot test assay for sub-MIC ceftaroline sensitivity. *S. aureus* CA-MRSA strain MW2 and derivatives with disruption in the TCS systems *srr* or *agr,* together with MW2 ΔXV, or ΔXIV versions (chromosomally complemented derivatives with the indicated TCS system in parenthesis). The upper panel corresponds to MHA plates containing 0.25 μg/mL of ceftaroline. Lower panel correspond to MHA plates without ceftaroline. Spot serial dilutions are indicated at the left margin. The first spot (10 μL) corresponds to 1.5 x 10^5 colony forming units (CFU). Note that disruption of neither *srr* nor *agr* results in a detectable change in viable colony counts at this sub-MIC ceftaroline concentration for these strains (MIC 0.5 μg/mL). Similarly, the restoration by chromosomal complementation of either *srr* or *agr* TCS system in the MW2 ΔXV strain does not detectably restore growth of the ΔXV strain, which is hypersensitive to ceftaroline in this condition.
